# STAT3在非小细胞肺癌耐药中的研究进展

**DOI:** 10.3779/j.issn.1009-3419.2019.07.08

**Published:** 2019-07-20

**Authors:** 思博 孙, 时代 金, 人花 郭

**Affiliations:** 210029 南京，南京医科大学第一附属医院肿瘤科 Department of Oncology, the First Afliated Hospital of Nanjing Medical University, Nanjing 210029, China

**Keywords:** 肺肿瘤, STAT3, 获得性耐药, Lung neoplasms, STAT3 transcription factor, Acquired drug resistance

## Abstract

近年来，肿瘤炎症微环境对非小细胞肺癌（non-small cell lung cancer, NSCLC）耐药影响的机制研究刚刚起步，信号传导及转录激活因子3（signal transducers and activators of transcription 3, STAT3）作为连接炎症和肿瘤的关键信号通路分子，其活化可引起肿瘤细胞中诸多基因沉默、表达异常及基因的不稳定等，诱导化疗、靶向药物治疗耐药，有望成为潜在的逆转耐药的新靶点。本综述阐述了STAT3在NSCLC获得性耐药中的研究进展，以探讨其作为逆转耐药新靶点的可能性，为NSCLC获得性耐药的临床治疗新策略提供理论依据。

## Abstract (en)

The inflammatory state of tumor microenvironment play an important role in non-small cell lung cancer (NSCLC) drug resistance. As the key signal pathway connecting inflammation and tumor, activated signal transduction and transcriptional activation factor 3 (STAT3) leads togenetic abnormal expression, gene silencing, genomic instability, *etc*. in tumor cells, and induces therapeutic resistance. STAT3 has thepotential to be a new target for reversal of resistance. In this review, we summarize the progress of STAT3 in acquired drug resistance of NSCLC, explore the possibility of STAT3 as a new target to reverse drug resistance, and provide basic theories for the new clinical treatment strategy of acquired drug resistance in NSCLC.

2018年世界卫生组织最新统计数据显示，全球肺癌新发病例接近210万，死亡病例约176万，肺癌已成为全球发病率及死亡率最高的恶性肿瘤^[1]^。而在中国，肺癌同样也是威胁人群健康的首要恶性肿瘤^[2]^。非小细胞肺癌（non-small cell lung cancer, NSCLC）为肺癌中最常见的类型，约占80%以上^[3]^。外科手术、化疗、放疗、靶向治疗及免疫治疗是目前主要的治疗手段，已在临床上广泛开展，也取得良好的疗效，但NSCLC患者5年生存率仍为10%-15%^[4]^。究其原因，获得性耐药是导致患者无法长期生存的主要原因之一。因此，深入研究NSCLC获得性耐药的机制，并籍此逆转耐药成为肺癌研究的热点与难点。

既往有关化疗、靶向治疗耐药的研究^[5-7]^大多为针对肿瘤细胞本身的耐药机制研究，获得性耐药问题尚不能得到有效解决。近年来研究^[8, 9]^发现肿瘤微环境在肿瘤耐药中发挥不可替代的作用。权威研究^[10, 11]^认为化疗及靶向治疗引起的肿瘤炎症微环境改变能够诱导肿瘤抵抗化疗和靶向治疗耐药。白细胞介素（interleukin 6, IL-6）作为肿瘤微环境中最关键的炎症因子之一，其相关IL-6R/JAK1/STAT3信号通路也在肿瘤的获得性耐药中发挥了重要的作用^[12]^。因此，本文着重介绍STAT3在NSCLC的化疗、靶向药物治疗及免疫治疗中的作用，初步阐述STAT3诱导NSCLC化疗及靶向药物耐药的机制，对STAT3作为NSCLC逆转耐药新靶点的可能性进行探讨。

## 1 STAT3信号通路

STAT3位于17q21染色体上，含24个外显子，DNA全长4, 815 bp，是一类由750个-800个氨基酸组成的DNA结合蛋白，分为α、β、γ 3种亚型^[13]^。STAT3由GP130受体家族激活，且IL-6家族通过STAT3的激活介导对细胞的调控。STAT3的激活主要包括赖氨酸乙酰化、丝氨酸磷酸化和酪氨酸磷酸化。其中酪氨酸磷酸化的STAT3可快速将细胞外信号传导至细胞内。磷酸化的STAT3首先在细胞质中形成同源二聚体，随后转位到细胞核中，结合DNA序列，调控整合基因的表达^[14, 15]^。STAT3可通过激活细胞周期进程相关的基因，如*cyclin* *D1*、*MYC*和*CDC25A*，参与细胞转化^[16, 17]^，此外还可诱导*BCL-2*、*BCL-XL*等生存基因及*MMP9*等侵袭转移相关基因的表达，进一步调控肿瘤细胞的增殖、分化、迁移^[18, 19]^。

STAT3是连接炎症和肿瘤的关键信号通路分子。肿瘤微环境中，炎症因子IL-6通过IL-6/JAK1/STAT3信号通路调控肿瘤细胞的发生发展。研究发现IL-6与GP130/IL-6受体复合物相应位点结合，从而诱导JAK1的激活，使STAT3磷酸化，磷酸化的STAT3与靶基因结合并修饰基因的表达，调控肿瘤的增殖、分化和凋亡^[12]^。同时，JAK1/STAT3通路为EGFR主要下游通路之一，EGFR通过激活STAT3，将信息传导至肿瘤细胞内，作用于靶基因，从而调控细胞增殖、分化与转移^[20]^。

## 2 STAT3对靶向药物耐药的影响

近年来，随着分子生物学和基因测序技术的发展，发现了越来越多的驱动基因。其中约40%-55%NSCLC患者发生表皮生长因子受体（epidermal growth factor receptor, *EGFR*）突变；其次是*KRAS*突变，占8%-10%；2%-3%的NSCLC患者存在间变性淋巴瘤激酶基因（anaplastic lymphoma kinase, *ALK*）融合突变。另有1%-2%的*ROS-1*重排；2%-3%的*RET*重排；2%-3%的*HER-2*突变等^[21]^。针对这些驱动基因的靶向药物也层出不穷，目前临床上应用最为广泛的靶向药为EGFR酪氨酸激酶受体抑制剂（tyrosine kinase inhibitor, TKI）及ALK-TKIs，他们分别是以吉非替尼、厄洛替尼为代表的一代EGFR-TKIs，以阿法替尼、达克替尼为代表的二代EGFR-TKIs，以奥希替尼为代表的三代EGFR-TKIs；以克唑替尼为代表的一代ALK-TKIs，以色瑞替尼、阿莱替尼为代表的二代ALK-TKIs以及以劳拉替尼为代表的三代*ALK-TKIs*。这些靶向药物在肺癌治疗中发挥了重要的作用，使NSCLC患者生存期明显延长，但获得性耐药的产生导致了肿瘤的进展与复发。目前关于EGFR及ALK酪氨酸激酶抑制剂的耐药的研究发现了*EGFR*及*ALK*位点的二次突变、旁路及下游通路的激活等机制^[6, 22]^，但这些针对肿瘤细胞自身的耐药机制研究尚不能完全解释获得性耐药的产生机制。研究发现，肿瘤细胞所处的肿瘤微环境中，炎症细胞通过分泌炎症因子IL-6，使STAT3的表达持续上调，可能为靶向药耐药的主要原因之一^[12, 23]^。

### 2.1 EGFR-TKIs

目前，EGFR-TKIs获得性耐药的主要机制有EGFR通路的二次突变，其中约50%的接受一代TKIs治疗的患者，通过第20号外显子T790M二次突变导致获得性耐药。其他机制包括旁路或下游通路激活，如*c-MET*扩增、*HER-2*突变、表型改变及肿瘤异质性等。仍有约30%的耐药机制不明^[24]^。

研究表明，在携带*EGFR* L858R和T790M突变的细胞中，STAT3抑制剂NSC74859可通过抑制EGFR下游传导通路JAK1/STAT3信号通路的激活来逆转吉非替尼及厄洛替尼耐药。同时，STAT3也参与了另一条EGFR下游通路AKT的激活，增强吉非替尼或厄洛替尼耐药性。其机制为持续EGFR-TKI的暴露抑制了AKT的上游调控因子PI3K的功能。通过STAT3的过表达取代了PI3K的作用，重新激活AKT/mTOR通路，从而诱导吉非替尼及厄洛替尼耐药^[23]^。除STAT3抑制剂外，一些具有抗肿瘤作用的天然化合物，如高三尖杉酯碱、多叶林I等，也可通过STAT3通路逆转吉非替尼耐药。在NCI-H1975细胞（*EGFR* T790M突变型吉非替尼耐药细胞株）中，高三尖杉酯碱通过抑制IL-6介导的IL-6R/JAK1/STAT3的STAT3 Y705磷酸化，进一步抑制STAT3向细胞核内转移。然后，通过抑制细胞核易位和转录活性，调控MCL-1和Survivin等凋亡蛋白的表达，诱导细胞凋亡，提高吉非替尼的敏感性^[25]^。多叶林I则是通过调节IL-6/STAT3信号通路，下调STAT3的表达，逆转上皮-间质转化（epithelial-mesenchymal transition, EMT）来克服厄洛替尼耐药^[26]^。

应激和缺氧等状态也可上调STAT3的表达，诱导获得性耐药的产生。高通量蛋白质组分析发现应激可激活NSCLC细胞上的β_2_-肾上腺素能受体，与EGFR形成协同信号，诱导肿瘤抑制因子肝激酶B_1_失活，进而诱导IL-6过表达，并通过IL-6/JAK1/STAT3信号通路，进一步上调STAT3的表达，抑制细胞对厄洛替尼的药物反应。结果显示，β受体阻滞剂联合IL-6抑制剂可逆转厄洛替尼耐药^[27]^。通过RNA测序技术，研究者们发现与吉非替尼单药组和单独低氧处理组相比，吉非替尼和缺氧联合处理组PC-9细胞的适应能力明显增强。其机制为，缺氧可刺激IL-6的产生，通过炎症相关肿瘤坏死因子，NF-κB及JAK信号转导因子的激活，刺激下游包括STAT3在内的转录信号通路富集，降低吉非替尼对PC-9细胞的作用，上述研究结果提示通过STAT3抑制剂联合吉非替尼逆转吉非替尼获得性耐药，在临床应用中有着广阔的前景^[28]^。

二代不可逆EGFR-TKIs（如阿法替尼、达克替尼等）获得性耐药的研究中发现STAT3可被IL-6自分泌和旁分泌机制进一步激活，诱导并增强获得性耐药。H1975和PC-9/GR（*EGFR* T790M突变型细胞）细胞可自分泌产生IL-6，激活IL-6/JAK1/STAT3信号通路，增强STAT3活化，诱导阿法替尼耐药。而H1975和PC-9/GR细胞与MRC5肺成纤细胞共培养后，通过旁分泌产生IL-6，耐药细胞表现出更强的STAT3的活化，导致其对阿法替尼产生的耐药性增强。而阻断IL-6/JAK1/STAT3信号通路后，STAT3表达下调，显著提高了共培养细胞对阿法替尼的敏感性。上述结果表明，自分泌与旁分泌IL-6R/JAK1/STAT3环在阿法替尼获得性耐药中起到了关键作用^[29]^。同样，NF-κB的激活，也可刺激细胞产生IL-6，并通过上述自分泌机制，使STAT3的表达上调，增强耐药性。同时，正因为STAT3不仅是IL-6的下游效应分子，也是IL-6活化的转录因子，形成了一个正反馈环，该正反馈过程进一步增强了阿法替尼的耐药性^[30, 31]^。研究发现，一种从植物中提取的生物碱——苦参碱，可通过降低H1579细胞中IL-6的表达，抑制JAK1/STAT3信号通路的激活，使STAT3表达下调，进而降低生存基因*Bcl2*的表达水平，增加阿法替尼药物敏感性，拮抗其获得性耐药。同样，小干扰RNA介导IL-6的表达下调，通过相同的机制，下调STAT3的表达，发挥抑制耐药性的作用^[32]^。同时，在阿法替尼耐药细胞中，IL-6受体抑制剂与β受体阻滞剂联用，可抑制STAT3的激活，逆转阿法替尼耐药。应激反应也可通过协同作用，使STAT3过表达，增加阿法替尼的耐药性^[27]^。

奥希替尼作为三代选择性不可逆EGFR-TKIs，其疗效得到了广泛的认可。FLAURA临床研究证明，与标准一线治疗相比，奥希替尼给NSCLC患者带来了更大的获益，特别是亚洲人群及脑转移的患者^[33]^。美国食品和药品管理局也批准奥希替尼作为*EGFR*第19外显子缺失或21外显子L858R突变型的NSCLC患者的一线治疗^[34]^。随着奥希替尼在临床的应用，耐药问题也随之出现。除了已知的Cys-797位点的突变，*MET*、*HER2*扩增及*PIK3CA*突变外^[35]^，经免疫接种产生的抗表皮生长因子抗体可通过抑制细胞外调节蛋白激酶1/2磷酸化和阻断STAT3的激活，下调受体酪氨酸激酶的表达，显著推迟了体外奥希替尼耐药的出现^[36]^，提示STAT3的激活可能与奥西替尼耐药密切相关。

### 2.2 ALK-TKIs

*ALK*融合突变多见于年轻、不吸烟人群。克唑替尼作为一代ALK-TKIs，可为*ALK*融合突变患者带来明显获益，平均无进展生存期可达6个月-10个月，近75%患者的总体生存期为1年^[37]^。二代ALK-TIKs阿莱替尼可使患者平均无进展生存期达到25.9个月^[38]^。2017年世界肺癌大会也报道了使用阿莱替尼后，无进展生存期超过4年的随访数据。尽管ALK-TKIs治疗疗效显著，如何解决其获得性耐药问题及如何进一步提高其疗效仍是肺癌研究的重点。目前已知的耐药机制可分为ALK依赖性和非ALK依赖性。ALK依赖性耐药机制主要包括二次突变、*ALK*基因拷贝数扩增等；非ALK依赖性耐药机制中，则为旁路的激活、肿瘤异质性及向小细胞肺癌的转化等^[39]^。

同时，STAT3也参与诱导了克唑替尼获得性耐药。STAT3可通过及EMT通路的上调诱导克唑替尼耐药。而STAT3抑制剂——Silibinin，可抑制STAT3与克唑替尼的相互作用，逆转耐药^[40]^。Silibinin亦可通过抑制EMT增强对克唑替尼的敏感性^[41]^。研究^[42]^表明，在H3122/TR（ALK抑制剂耐药的细胞）细胞中，EML4-ALK的表达降低，而EGFR、HER2、HER3的磷酸化水平升高，并伴有表皮生长因子分泌的增加，进而诱导STAT3磷酸化。结果表明，STAT3参与的ErbB受体家族的激活与肺癌*ALK-EML4*融合患者ALK-TKIs耐药有关。

### 2.3 其他靶向药物

除常见的EGFR-TKIs与ALK-TKIs外，STAT3也参与了其他靶向药物的耐药。司美替尼（AZD6244）是一类针对KRAS、ALK、MET及HER-2的多靶点的MEK抑制剂。在司美替尼耐药的结肠癌细胞中发现，JAK2-STAT3信号表达上调。司美替尼联合JAK2/STAT3抑制剂AG490使用可显著抑制细胞增殖，诱导细胞凋亡，完全抑制ERK和JAK2/STAT3信号的激活，下调STAT3的表达^[43]^。那么，在NSCLC中，司美替尼获得性耐药是否与JAK2/STAT3通路的激活有关，还需更多的实验来进一步探究。

## 3 STAT3对化疗耐药的影响

化疗是治疗NSCLC的基石。ECOG1594研究所确立的含铂双药化疗仍然是晚期NSCLC的标准一线治疗，可使患者中位生存期达8个月左右^[44]^。然而，化疗耐药极大的限制了NSCLC治疗的有效性。目前研究表明，除各种原因所致的化疗药物在肿瘤细胞中药物浓度的减少而产生的化疗耐药外^[5]^，研究发现STAT3在化疗耐药中也发挥了重要的作用。在顺铂耐药的NSCLC中，整合素-金属蛋白酶8过表达，且STAT3的表达量显著增加。而RNA干扰沉默STAT3后，其下游*Bcl*‐*2*和*Mcl*‐*1*细胞生存基因的表达明显下调，部分逆转了顺铂获得性耐药。因此，整合素-金属蛋白酶8通过激活STAT3信号通路参与了NSCLC细胞对顺铂的耐药^[45]^。除了整合素-金属蛋白酶8的过表达可上调STAT3水平，诱导耐药外，另有研究^[46]^证明，多种致癌基因的过表达，如*Bcl-2*、*c-Myc*、*cyclin D1*，经miR-197/CKS1B/ STAT3信号通路调控肿瘤发生发展，抑制顺铂耐药。同时，有研究^[47, 48]^发现，STAT3 mRNA在顺铂耐药NSCLC细胞中过表达。结果表明，STAT3在转录水平表达的上调可能抑制细胞凋亡途径，诱导顺铂耐药。而毛细血管扩张突变也在NSCLC顺铂耐药细胞中高表达。其高表达可通过IL-6R/JAK1/STAT3信号通路激活NSCLC细胞的EMT中twist、snail、slug、foxc2、zeb1和zeb2等进程，诱导EMT产生，进一步增强顺铂耐药性^[49]^。

在肿瘤微环境中，肿瘤细胞、癌相关成纤维细胞、内皮细胞、免疫细胞等通过炎症因子相互作用。而癌相关成纤细胞与肿瘤细胞通过IL-6的自分泌环相互作用，诱导并经一步增强IL-6R/JAK1/STAT3信号通路的激活，使STAT3表达上调，导致化疗耐药。同时，肿瘤细胞产生的TGF-β与癌相关成纤细胞可能够增强肿瘤细胞的EMT，而炎症因子IL-6可通过IL-6R/JAK1/STAT3信号通路，进一步增强STAT3的激活，诱导化疗耐药^[50]^。

STAT3不仅参与了NSCLC顺铂获得性耐药，研究者们还发现小干扰RNA沉默STAT3使NSCLC耐药细胞对紫杉醇及阿霉素更加敏感。同时，他们发现STAT3主要是通过调控促生存通路来调节对细胞毒药物的敏感性，而其不是通过对肺癌细胞的增殖和凋亡的控制来发挥作用^[51, 52]^。

## 4 STAT3对免疫治疗的影响

免疫检查点，如程序性细胞死亡蛋白-1（programmed death-1, PD-1）、程序性死亡受体-配体（programmed death-ligand 1, PD-L1），是免疫系统中一些负性调节信号通路。在肿瘤发生时，免疫检查点的激活可抑制自身免疫，使肿瘤发生免疫逃逸。PD-1主要作用于效应T细胞，活化的T细胞、B细胞及NK细胞均可诱导PD-1的产生。PD-1与PD-L1或PD-L2结合后，介导T细胞活化的共抑制信号，抑制T细胞的杀伤功能^[53]^。免疫检查点抑制剂可阻断这些负性调节信号通路，通过逆转肿瘤微环境，发挥抗肿瘤作用^[54]^。抗PD-1/PD-L1抗体作为最常见的免疫检查点抑制剂现已在临床中得到较为广泛的应用。最初只有少部分患者可从免疫治疗明显获益，大多数患者仅表现为部分有效。尽管部分患者开始可获得良好的疗效，但越来越多的患者在免疫治疗期间出现病情进展，产生耐药。根据其耐药机制，可分为原发性耐药、适应性耐药及获得性耐药^[55]^。肿瘤微环境中抑制性免疫检查点（如调节性T细胞、髓源抑制性细胞）的募集，可通过直接接触或分泌抑制性细胞因子（如IL-10），抑制T细胞增殖、细胞因子生成及降低细胞毒性，来抑制效应T细胞的杀伤功能，介导免疫逃逸，产生原发性和适应性耐药^[56]^。

STAT3作为细胞因子IL-6下游关键转导因子，其异常表达可能与免疫治疗耐药有关。不仅如此，STAT3还参与调控了PD-L1的表达。CheckMate 057与KEYNOTE-010临床研究分析了二、三线NSCLC患者接受PD-L1/PD-1抑制剂治疗的疗效，结果表明PD-L1的表达越高，疗效越好^[57, 58]^。既往研究^[59]^显示，STAT3可直接与PD-L1启动子结合，在转录水平上调控PD-L1的表达。在抗原提呈细胞中，阻断p38和p44/42 MAPKs可降低IL-6的表达水平，进而抑制STAT3的活化，下调PD-L1的表达。AKT/STAT3信号通路的抑制，可降低NSCLC细胞中PD-L1的表达。而PD-L1表达的沉默可抑制NSCLC细胞的增殖，诱导NSCLC细胞的凋亡^[60]^。胸腺肽1通过下调STAT3-MMP2信号通路，显著抑制PD-L1高表达的NSCLC细胞的迁移和侵袭^[61]^。而JAK/STAT信号传导通路在免疫应答过程中也发挥重要的作用^[62]^。综合上述研究，STAT3通路的激活可使PD-L1的表达上调，从而增强细胞的增殖、迁移和侵袭能力，并抑制细胞的凋亡能力。一项临床研究^[63]^发现，由于缺乏PD-L1的表达，恶性黑色素瘤及结肠癌细胞中JAK1/2功能缺失突变介导了对PD-1/PD-L1免疫治疗的耐药性。而STAT3作为该通路下游重要的信号因子，其在NSCLC中是否也参与了免疫耐药值得进一步的探究。

## 5 PD-L1通过STAT3参与靶向药物及化疗耐药

PD-L1的表达是一个动态变化过程，其表达随着治疗及耐药的产生而发生改变。研究^[64]^发现，STAT3表达的上调，可诱导PD-L1的表达，且JAK/STAT3通路与肿瘤的免疫逃逸高度相关，提示PD-L1表达的升高引起的肿瘤免疫逃逸可导致耐药的出现。

活化的EGFR可通过激活IL-6/JAK/STAT3信号通路，使STAT3表达上调，诱导PD-L1的表达。在吉非替尼治疗后，STAT3的表达下调，抑制了PD-L1的表达，进一步验证了这一结果^[65]^。另外，在吉非替尼耐药细胞中，抑制AKT/STAT3信号通路同样可通过下调STAT3的表达，使PD-L1的表达下调^[66]^。在克唑替尼耐药的NSCLC细胞中，STAT3的激活亦可上调PD-L1的表达^[40]^。同样，在化疗耐药中，PD-L1的表达也通过STAT3的激活上调。研究发现，在NSCLC顺铂耐药细胞中可检测出毛细血管扩张突变，JAK及STAT3高表达。毛细血管扩张突变可通过激活JAK/STAT3信号通路诱导STAT3过表达，进而上调肿瘤细胞中的PD-L1的表达，诱导耐药。而毛细血管扩张突变被特异性抑制剂作用后，表达下调，进而抑制STAT3的激活，下调耐药细胞的PD-L1的表达^[49]^。microRNA-3127-5p可通过抑制自噬，促进STAT3磷酸化，上调PD-L1的表达。而敲除miRNA-3127-5p后，自噬可将pSTAT3保留在细胞核内，无法进一步诱导PD-L1的表达，降低了化疗耐药性^[67]^。提示PD-L1的过表达与靶向药物及化疗获得性耐药有关。而STAT3可作为调控PD-L1异常表达的潜在治疗靶点，逆转化疗及靶向治疗耐药。

## 6 总结与展望

STAT3信号转导通路是包括NSCLC在内的多种恶性肿瘤产生获得性耐药的重要途径。大量体内外实验中得出IL-6R/JAK1/STAT3信号通路抑制剂单药或联合其他抗肿瘤治疗可逆转化疗，靶向治疗获得性耐药及放疗抵抗。缺氧及应激反应，可通过活化STAT3，调控下游*BCL*-*2*等基因的表达，增强细胞对药物的耐药性。目前，多种STAT3抑制剂及抗IL-6靶向药的临床研究已在进行中，但这些研究均不包括NSCLC治疗耐药患者^[68, 69]^，所以我们期待更多的临床研究来进一步验证STAT3信号通路是否可作为NSCLC获得性耐药的治疗新靶点。

STAT3的激活可上调顺铂、吉非替尼及克唑替尼耐药细胞中PD-L1的表达。提示STAT3的激活并上调PD-L1的表达可能是NSCLC获得性耐药的重要机制之一，且STAT3可能作为临床中治疗获得性耐药的潜在靶点。那么STAT3抑制剂联合免疫治疗是否可逆转化疗，靶向治疗获得性耐药，是否可在临床中发挥作用，还有待相关研究进一步验证。

综上所述，STAT3参与了NSCLC的靶向药物及化疗耐药，可能与免疫治疗的获得性耐药有关。其可能作为NSCLC获得性耐药逆转耐药的潜在治疗靶点，为临床NSCLC获得性耐药治疗提供新策略。
